# Antisepsis before skin injections: does the WHO recommendation for washing, instead alcohol-based antisepsis, achieve the same efficacy?

**DOI:** 10.3205/dgkh000581

**Published:** 2025-09-22

**Authors:** Torsten Koburger-Janssen, Paula Zwicker, Ojan Assadian, Martin Exner, Uche Eze, Jürgen Gebel, David Leaper, Simone Scheithauer, Miranda Suchomel, Axel Kramer

**Affiliations:** 1 Hygiene Nord GmbH, Greifswald, Germany; 2 Institute of Hygiene and Environmental Medicine, University Medicine Greifswald, Germany; 3 Section Antiseptic Stewardship of the German Society of General and Hospital Hygiene; 4 University Hospital Wiener Neustadt, Wiener Neustadt, Austria and Danube Private University, Wiener Neustadt, Austria; 5 Emeritus Professor, Institute of Hygiene and Public Health, University of Bonn, Germany; 6 Department of Clinical Pharmacy and Pharmacy Management, University of Nigeria, Nsukka, Nigeria; 7 Institute of Hygiene and Public Health, University of Bonn, Germany; 8 Emeritus Professor, Universities of Newcastle and Huddersfield, UK; 9 Department of Infection Control and Infectious Diseases, University Medical Center Göttingen (UMG), Georg-August University Göttingen, Göttingen, Germany; 10 Institute of Hygiene and Applied Immunology, Medical University of Vienna, Vienna, Austria

**Keywords:** infection risk skin injections, skin antisepsis, efficacy soap washing, efficacy alcohol-based antiseptics

## Abstract

**Introduction:**

Cleaning and reduction of microbial load on the skin is necessary before injections because of the colonization of the skin with resident flora and the presence of transient flora. Even after optimal skin antisepsis, there is a risk of infection, albeit a very low one.

The World Health Organisation (WHO) based on a systematic review on the infection risk after injections and skin antisepsis, does not recommend, alcohol-based skin antisepsis as being necessary before intradermal, subcutaneous and intramuscular injections and that washing the skin with soap and water alone is sufficient. As there is no clear evidence for the benefit of using alcohol over soap washing prior to injection, this study compared the efficacy of alcohol and soap on volunteers.

**Method:**

Liquid washing emulsion or potash soap (Sapo Kalinus, German Pharmacopoeia) or propan-2-ol 70%v/v, was applied to test areas on the upper arms of 23 volunteers. To test the soap, in trial 1 after 15-second swabbing, and a further 15 seconds of air-drying, the swabbed areas were rinsed, dabbed dry, and microbiological samples taken with sterile swabs. In trial 2, after 15 seconds of swabbing, samples were taken in the same manner. The comparator alcohol was rubbed in for 15 seconds in trial 1 and for 30 seconds in trial 2 without rinsing. Swabs were transferred into tryptic soy broth and suspensions plated onto agar.

**Results:**

The reduction of bacteria was around 1x lg and did not differ significantly between the soaps and propan-2-ol. There was also no difference when comparing the two trials.

**Discussion:**

One reason for the equivalent efficacy of both methods is probably the low colonization density of the skin of the arms.

**Conclusion:**

Both methods are acceptable for legal reasons. However, if no live vaccines are applied, alcohol-based antiseptics are preferable because they are more user-friendly.

## Zusammenfassung

**Einleitung:**

Auf Grund der Kolonisation der Haut mit residenter Flora und dem Vorkommen von transienter Flora ist vor Injektionen die Reinigung und Keimzahlverminderung erforderlich. Das wird dadurch bestätigt, dass trotz vorheriger Hautantiseptik ein, wenn auch sehr geringes, Infektionsrisiko besteht.

Im Ergebnis der Literaturrecherche zum Infektionsrisiko nach Injektionen und zu Empfehlungen zur Hautantiseptik stellte sich heraus, dass die WHO die Alkohol-basierte Hautantisepsis vor intradermalen, subkutanen und intramuskulären Injektionen nicht für erforderlich hält und empfiehlt, die Haut mit Wasser und Seife zu waschen. Da es keine epidemiologischen Beweise für die Hautantisepsis mit Alkohol gibt, sollte die Wirksamkeit von Alkohol und Seife bei Freiwilligen verglichen werden.

**Methode:**

Auf Testflächen an den Oberarmen von 23 Freiwilligen wurden eine flüssige Waschemulsion, Sapo Kalinus oder Propan-2-ol 70% v/v aufgetragen. Um die Seife zu testen, wurden in Versuch 1 nach 15 s Abstrich und nach weiteren 15 s Lufttrocknung die abgetupften Stellen abgespült, trocken getupft und mit sterilen Tupfern Proben entnommen. In Versuch 2 wurden die Proben nach 15 s Abstrich auf die gleiche Weise entnommen.

Der Alkohol wurde in Versuch 1 15 s und in Versuch 2 30 s lang eingerieben, ohne dass gespült wurde. Die Abstriche wurden in Tryptose-Soja-Bouillon überführt und die Suspensionen auf Agar ausplattiert.

**Ergebnisse:**

Die Reduktion lag bei etwa 1 lg und unterschied sich nicht signifikant zwischen Seife und Propan-2-ol. Auch beim Vergleich der beiden Versuche gab es keinen Unterschied.

**Diskussion:**

Ein Grund für die gleichwertige Wirksamkeit beider Methoden ist möglicherweise die geringe Besiedlungsdichte der Haut an den Armen, womit ein statistischer Unterschied unterhalb der Nachweisgrenze liegen könnte.

**Schlussfolgerung:**

Beide Methoden sind aus experimentellen Gründen akzeptabel. Wenn keine Lebendimpfstoffe appliziert werden, sind jedoch bei Verfügbarkeit Antiseptika auf Alkoholbasis vorzuziehen, da ihre Anwendung benutzerfreundlicher ist.

## Introduction

At present, there is no clear evidence base for the necessity of alcohol-based skin antisepsis before skin injection. Only one randomised controlled trial (RCT) has studied skin injection, with or without prior antisepsis with 70% propan-2-ol and the effect on skin microbiology, but it was underpowered with 170 children [1]. Based on the results of another, three-arm randomized study including 450 included children [2], a review [3] concluded that it might not be necessary to use skin antiseptics for routine vaccinations or injections.

The Forum for Injection Technique, UK [4], the Australian Department of Health [5] and the Forum for Injection Technique and Therapy Expert Recommendations, India [6], also do not consider skin antisepsis necessary before subcutaneous injection of insulin or vaccines. Six studies suggest that there is no increased risk of infection after insulin injection without prior application of skin antiseptics (cited in [7]). At the Expert Recommendations Forum on Injection Technique and Therapy, 183 diabetes experts from 54 countries formulated the following recommendations [8]:

- Patients should inspect the skin site before injection. Injections should be given into clean sites, using clean hands.
- If the site is found to be unclean it should be disinfected. Disinfection is also required in institutional settings such as hospitals and nursing homes. If alcohol is used, it must be allowed to dry completely before the injection is given.
- Antisepsis is usually not required when injections are given in non-institutional settings such as homes, restaurants, and workplaces.
- Patients should never inject into sites of skin diseases, inflammation, oedema, ulceration, or clinical infection.
- Patients should not inject through clothing because they cannot inspect the site beforehand or easily lift a skinfold.

The World Health Organization (WHO) suggests that, in general, alcohol-based skin antisepsis is not necessary before intradermal, subcutaneous and intramuscular injections, and recommends washing the skin with soap and water alone [9].

In contrast, in the German guideline on hygiene requirements for punctures and injections, alcohol-based skin antiseptics are recommended before any skin injection [10]. The Public Health Agency of Canada [11] also advises the practice of treating skin with a suitable antiseptic solution prior to vaccination or injection. Similarly, the Nigeria Centre for Disease Control for preparing and administering injections has recommended the importance of antiseptic measures for their country [12], and alcohol-soaked swabs or cotton wool is the standard practice for skin preparation. A study conducted at the Federal Medical Center, Yanagoa, Bayelsa reported that in Nigeria, healthcare workers usually used cotton balls soaked in alcohol to decontaminate skin prior to injection [13]. Certified infection control nurses in Japan perform skin antisepsis before subcutaneous injection in hospitals in accordance with their hospital standards but outside the hospital, subcutaneous injections are administered without skin antisepsis [14].

However, it has to be mentioned that in some cases an alternative method, as cleaning of the skin with soap and water before injection may be practiced also in hospital settings and medical practices, which in most cases is influenced by resource availability. Although alcohol has been permitted for medical use, not all health care workers feel comfortable with using alcohol, as they fear inhalation or absorption through skin [15]. Although it is recommended to wait until skin is completely dry after alcoholic antisepsis, before administration of smallpox vaccine, alcohol-based skin antiseptics should be avoided, because alcohol residues can inactivate the vaccinia virus [16]. Similar precautions should be made for attenuated vaccines such as mumps, measles, rubella, dengue or yellow fever. 

Interestingly, in a questionnaire at Penang General Hospital, Malaysia, >90% of healthcare professionals stated that alcohol swabbing of injection sites prevent infection and did not believe that cleaning with water and soap is sufficient [17].

To clarify whether skin cleaning prior to vaccination can reduce local skin infections, and if skin antisepsis is superior to soap and water alone, further research is needed. Moreover, studies to examine other types of injections will also be able to determine whether skin preparation is necessary for all injections [3]. To assess clinical evidence of the post-injection infection rate after subcutaneous, intradermal or intramuscular injection (Table 1 (Tab. 1)), the sample size must be increased to >100,000 per group to show a difference. 

The aim of this preliminary study was to determine the efficacy of skin antiseptics using propan-2-ol 70% v/v compared with soap using an accepted model of microbial decontamination for testing skin antiseptics on volunteers. This could give the basis for further research such as determination of sample size in an RCT. A cross-over design with 23 volunteers was used to assess the bacterial reduction following skin preparation with 70% v/v propan-2-ol, or two soaps: liquid washing emulsion seba med^®^ FLÜSSIG WASCH-EMULSION, and (a potash soap, sapo kalinus, German Pharmacopoeia).

## Methods

### Requirements

According to the German “Verbund fur Angewandte Hygiene e.V.” (VAH; Association of Applied Hygiene) test method 13 for certification of skin antiseptics in Germany [18], the test was performed on the upper arm of volunteers. The exclusion criteria were skin not treated with disinfectants or antiseptic solutions within three days prior to the test; no antibiotic therapy before the test because of possibly altered skin flora; dermatoses in the test area and fever. The inclusion criteria were complied with and informed signed consent was undertaken to participate in the study.

The study was conducted in compliance with the World Medical Association (WMA) Declaration of Helsinki, Ethical Principles for Medical Research [19], the State Data Protection Act and the General Data Protection Regulation as well as the Professional Code of Conduct for Physicians in Mecklenburg Western Pomerania [20]. In addition, the statement from the Federal Institute for Drugs and Medical Devices of Germany (BfArM) [21] affirms “that testing of chemical disinfectants and antiseptics phase 2, step 2, according to the European Norm EN 12791 [22], are explicitly not treated by the BfArM as clinical trials within the meaning of the Medicinal Products Act. This means that the requirements for pharmacological monitoring required for clinical trials and the fulfillment of other requirements in accordance with good clinical practice are not necessary.”

The study was conducted in November 2023 (trial 1) and repeated in March 2024 (trial 2) with other volunteers to confirm the results. 

### Study design

**Figure 1:**
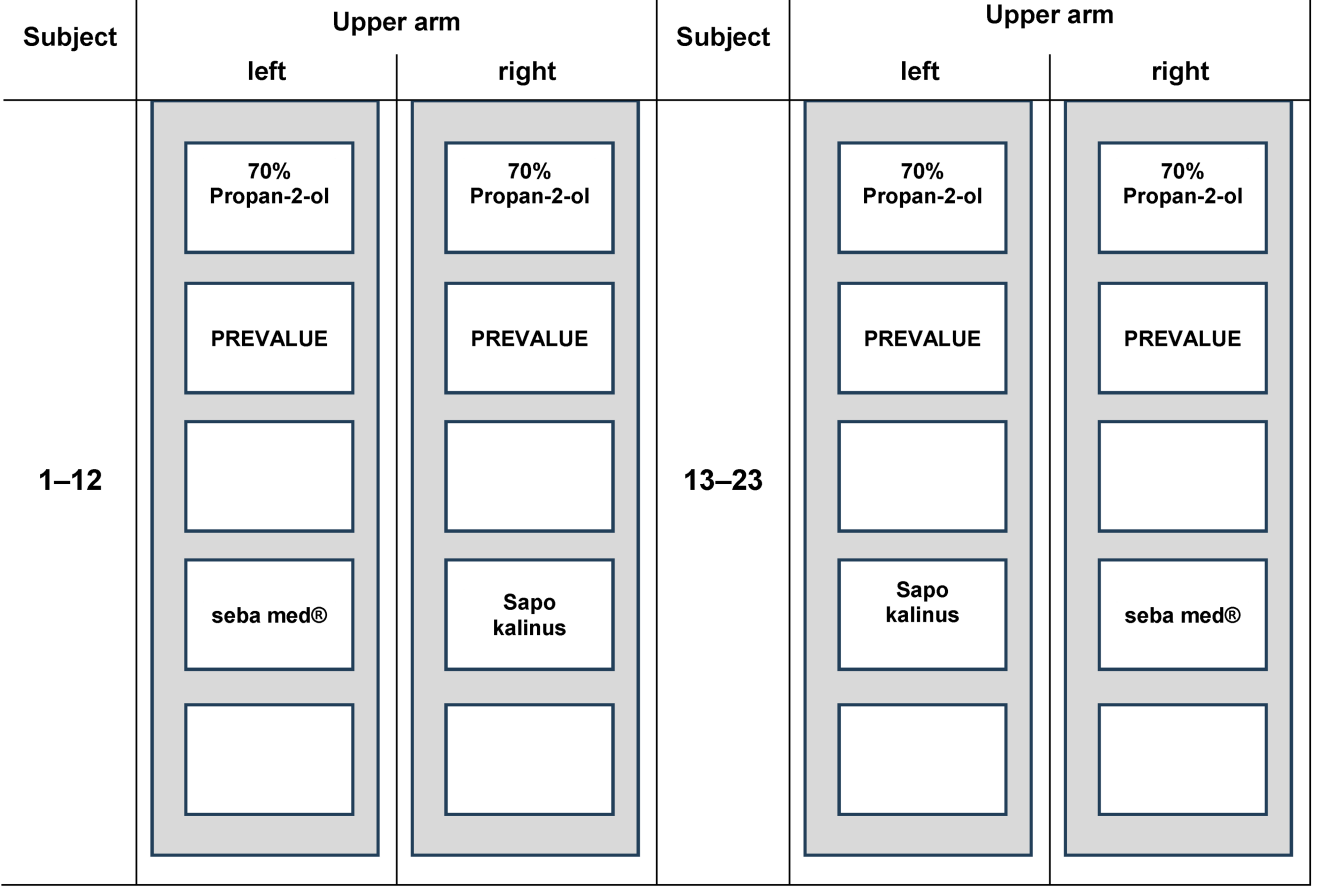
Distribution of the test areas on the volunteers’ upper arm (each test area with the required dimensions of 5 cm^2^)

A cross-over design was used with 23 volunteers (13 female, 10 male, age between 23 and 72; mean 440.49, white-skinned) relation to the right and left arm on marked test areas (Figure 1 (Fig. 1)). Comparison was made between 70% v/v propan-2-ol (Carl Roth GmbH + Co. KG, Germany), which is the reference product of the German test method [18] and two soaps, S1=liquid washing emulsion seba med^®^ FLÜSSIG WASCH-EMULSION (Sebapharma GmbH & Co. KG, Germany) and S2=Sapo Kalinus (Pielsegura Cosmética Artesana S.L Molonicos/Albacete, Spain), which is the reference product of EN 1499 for hygienic hand wash [23]. 

### Determination of pre-treatment values

The pre-treatment value was determined in the first test field on each arm: Using a cotton swab moistened in 5 ml tryptic soy broth (TSB, Carl Roth GmbH & Co. KG Karlsruhe, Germany) with neutralizing agent A (Table 2 (Tab. 2)), the marked test field was thoroughly swabbed for 15 seconds. Care was taken to ensure that the swab did not go beyond the edges.

The swabs were then transferred to 5 ml TSB/neutralizer A and shaken for 30 s at high frequency in a test tube shaker. This collection liquid was further diluted 1:10 in TSB/neutralizer. Of undiluted and diluted sample, 0.1 ml were plated onto tryptic soy agar plates (TSA, Carl Roth GmbH + Co. KG Karlsruhe, Germany) and cultivated for 48 h at 36±1°C.

### Treatment with reference and test products

Propan-2-ol (70% v/v), the reference product, was applied using VAH 13 standard cotton swab stick [18]. The exposure times for the reference procedure were 15 s for test 1 and 30 s for test 2. One arm was treated with the reference product, the other with the test product (S1 or S2). 

The test products (S1, S2) were applied by using sterile gauze balls (Fuhrmann GmbH; “Schlinggazetupfer”, Germany). For application, the gauze balls were soaked in a Petri dish with 3 ml of water of standardized hardness (WSH), as this volume avoids excess spillage of liquid during application. After a soaking time of 2 min, 1 ml of the test product was added to the upper side of the soaked gauze ball. For the scrub, the gauze ball was picked up with gloved hands and used for repeatedly wiping up and down the test side with an application area of about 8x3 cm for 15 seconds. The standard 2.5x2 cm sampling template was then placed over this area. Pressure was firmly applied to the test volunteer’s skin without damaging the skin. Two trials were performed:

In trial 1 after 15-second swabbing, and after further 15 seconds of air drying, the swabbed area was rinsed with 20 ml WSH using a sterile 25 ml syringe. Finally, the area was dabbed dry with a sterile paper towel and the sample was taken with a sterile swab in the centre of the application area using a positioning template. 

In trial 2, immediately after 15 seconds of swabbing, the samples were taken in the same manner as in trial 1.

The swabs were transferred into 5 ml TSB containing neutralizing agent A or B (Table 2 (Tab. 2)) and shaken for 30 s at high frequency in a test tube shaker. From these samples a 1 to 10 dilution was made. Undiluted and diluted samples (0.1 ml) were plated onto TSA plates and cultivated for 48 hours at 36±1°C. 

To avoid any interference between the test sites, the pre-values were determined first, followed by the application and sampling of the test products and, finally, the reference testing undertaken with propan-2-ol. 

### Calculation of reduction

The calculation was performed according to EN DIN 12791 [22]. After counting the plates, the colony forming units (cfu) per 1 ml were converted into decade logarithms. The reduction (R) was calculated using the following formula: 

R=lg(pre-value)–lg(post-value)

For the statistical calculation, the Wilcoxon signed-ranks test was used to compare the 15 s reduction of the reference and test products. Finally, the results of trial 1 and 2 were checked for consistency. Due to the predominantly exploratory nature of the testing, the significance level was set at p=0.1. A one-sided test was performed.

## Results

The reduction ranged around 1 lg (Table 3 (Tab. 3)) and did not differ significantly between the soaps and propan-2-ol in trial 1 and 2. There was also no difference when comparing the two trials (p>0.5).

## Discussion

Antisepsis of injection sites using alcohol-based antiseptics is common in clinical practice. However, it is not required universally and infection rates are low (Table 1 (Tab. 1)). However, antisepsis in clinical situations provides additional safety and is easier to perform compared to washing with soap and water. In cases where alcohol-based antiseptics are not feasible such as administration of certain vaccines like smallpox vaccines, alcohol residues could inactivate the vaccinia virus, which may also occur when other vaccines such as mumps, measles, rubella, dengue or yellow fever are being used [16] or when alcohol-based antiseptics are simply not available, washing with soap and water is an alternative that should be considered.

In case of vaccination, it is recommended to wait until the skin is completely dry and probably alcohol alone would not interfere with the vaccine, but the risk may exist if the alcoholic disinfectant contains a persisting active agent such as chlorhexidine. The Nigerian Expanded Program on Immunization discourages the use of antiseptics when live vaccines are being administered. Instead, healthcare providers are recommended to use swabs soaked in clean water for the preparation of the injection site. An analysis of data showed that, during the follow-up assessment, 78% of 236 observations confirmed the practice of cleansing the patient's skin before administering vaccinations. This shows a decrease from the baseline assessment, where 7 out of 8 observations maintained the practice of soaking the swab in clean water to clean the injection site prior to vaccination. This outcome underscores the necessity for training and enhancement in healthcare provider practices in this specific domain [24].

The test design used aimed to imitate practical conditions as far as possible. The upper arm simulates practical conditions for skin injections in areas, which contain few sebaceous glands. Since in practice a few seconds often elapse between swabbing and injection when skin antisepsis is performed, in addition to sampling immediately after the prescribed exposure time of 15 seconds sampling (trial 2), a 15-second air-drying time after 15 seconds of swabbing was allowed to elapse before sampling (trial 1).

Other antiseptic agents, such as alcoholic or aqueous solutions of chlorhexidine digluconate (CHG), povidone-iodine (PVP-I), or alcohols without addition of a further antiseptic agent can be used. Alcoholic solutions are superior to non-alcoholic solutions in regard to prevention of blood culture contamination after venous puncture [25]. Calfee and Farr concluded in their study that, propan-2-ol may be the optimal antiseptic, given its convenience, low cost, and tolerability [26]. In the present study no statistically significant differences between use of soap washing or antisepsis using propan-2-ol was found in antibacterial efficacy. The results between trial 1 and 2 did not differ, which suggests that the equivalence of skin cleansing with soap or alcohol-based skin antisepsis is equivalent if the injection is carried out later after skin antisepsis.

One reason for the equivalent efficacy of the two methods, swabbing with alcohol or soap, is probably the low colonization density of the skin of the arms, with 10^2^–10^3^ cfu/cm^2^ [27]. Since we determined those low colony counts in the pre-values, reaching a lg reduction using propan-2-ol or soap might also be the maximum reduction that can be achieved. However, a lg reduction of >1 indicates inactivation of >90% of bacteria indicating that soap washing is a good alternative measure to alcoholic disinfection for infection prevention. One limitation of the study is its small sample size.

Comparison with other studies is difficult because few of them measured antibacterial efficacy of antiseptics after short contact times of <1 min. The data published by Reichel et al. are comparable to ours [28]. They stated a lg reduction of 1.21±0.65 to 2.43±0.72 after 1 min contact time to propan-2-ol at different body sites (forehead, upper back, abdomen and lumbar area [28]. 

Comparing results of bacterial removal following washing with soap and water to those of propan-2-ol antisepsis, soap washing is not as user-friendly as the use of alcoholic antiseptics since it requires clean running water, longer drying time, carries the risk of secondary contamination of the soap and intrinsic contamination of the water used for rinsing, as well as transmission of soap residues through the skin. In contrast, the proven microbicidal efficacy of alcohol-based skin antiseptics guarantees approved efficacy and safety, and the application technique can be better standardized. Since alcohol-based skin antisepsis does not affect blood alcohol content [29], this is not an argument against the use of alcohol-based skin antiseptics. 

The efficacy of soap and water is based solely on the mechanical removal of pathogens, whereas an alcohol-based antiseptic is a combination of microbicidal action and mechanical removal; the latter achieves a higher level of safety. This can be relevant in the event of skin contamination with highly virulent bacteria such as beta-hemolytic streptococci. If skin antisepsis is not performed prior to an injection, the question arises as to whether a healthcare provider should ensure that no pathogenic bacteria are present prior to injection. In Germany, compensation for pain and suffering in the amount of €10,000 was granted following a liability lawsuit for failure to perform skin antisepsis prior to injection and the resulting infection [30]. Lawrence et al. [31] point out the problem of determining the burden of proof in the case of infections which follow omission of skin antisepsis.

## Conclusion

With the test model on the upper arm, swabbing with liquid soap or alcohol-based antiseptic were equally effective. Even if low sample size limits the significance of the study, the data indicate that both application options are acceptable for legal reasons. Due to the theoretically higher safety level, the independence of access to running microbiologically safe tap water and the simpler application, alcohol-based antiseptics are preferable if no live vaccines are to be used.

## Notes

### Competing interests

The authors declare that they have no competing interests.

### Funding

The testing was financially supported by the Association of Applied Hygiene, Germany.

### Author contributions

The authors Koburger-Janssen and Zwicker contributed equally.

### Authors’ ORCIDs

- Kramer A: https://orcid.org/0000-0003-4193-2149
- Zwicker P: https://orcid.org/0000-0001-8891-7160
- Assadian O: https://orcid.org/0000-0003-0129-8761
- Eze U: https://orcid.org/0009-0002-8702-4357
- Gebel J: https://orcid.org/0000-0001-9328-3174
- Leaper D: https://orcid.org/0000-0002-6662-9712
- Scheithauer S: https://orcid.org/0000-0003-0773-4739
- Suchomel M: https://orcid.org/0000-0001-8758-9652

### Ethical approval 

The study was conducted in compliance with the WMA Declaration of Helsinki, Ethical Principles for Medical Research, the State Data Protection Act and the General Data Protection Regulation, with the Professional Code of Conduct for Physicians in Mecklenburg Western Pomerania and with the statement from the Federal Institute for Drugs and Medical Devices of Germany (BfArM). 

## Figures and Tables

**Table 1:**
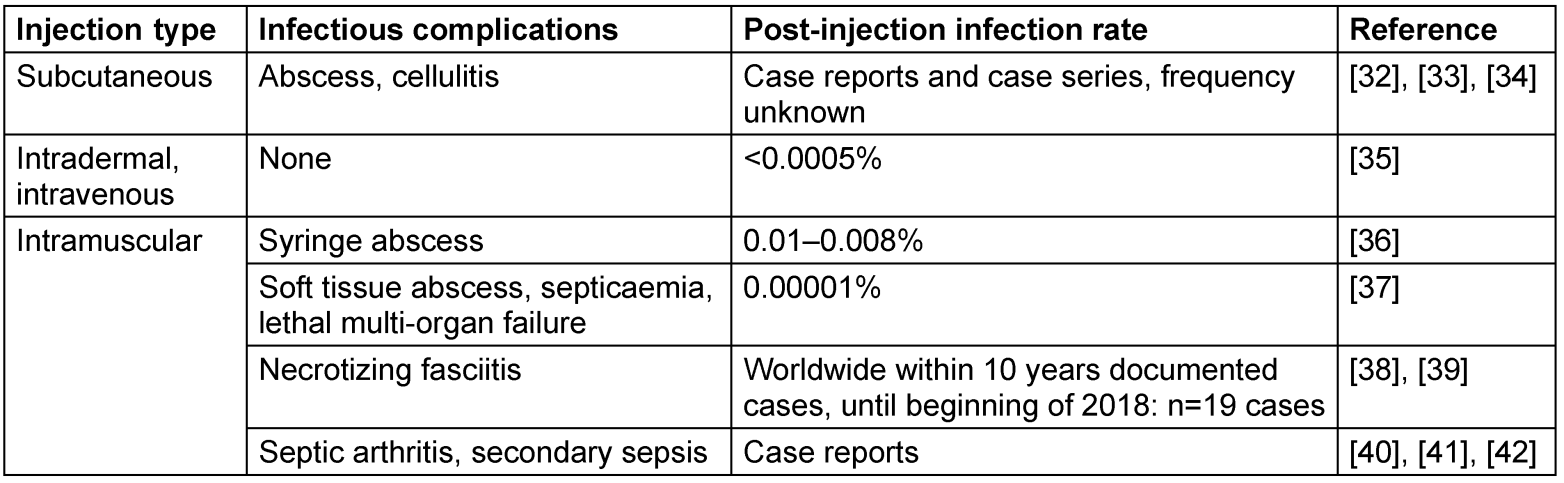
Published post-injection infection rates and infectious complications following skin antisepsis

**Table 2:**
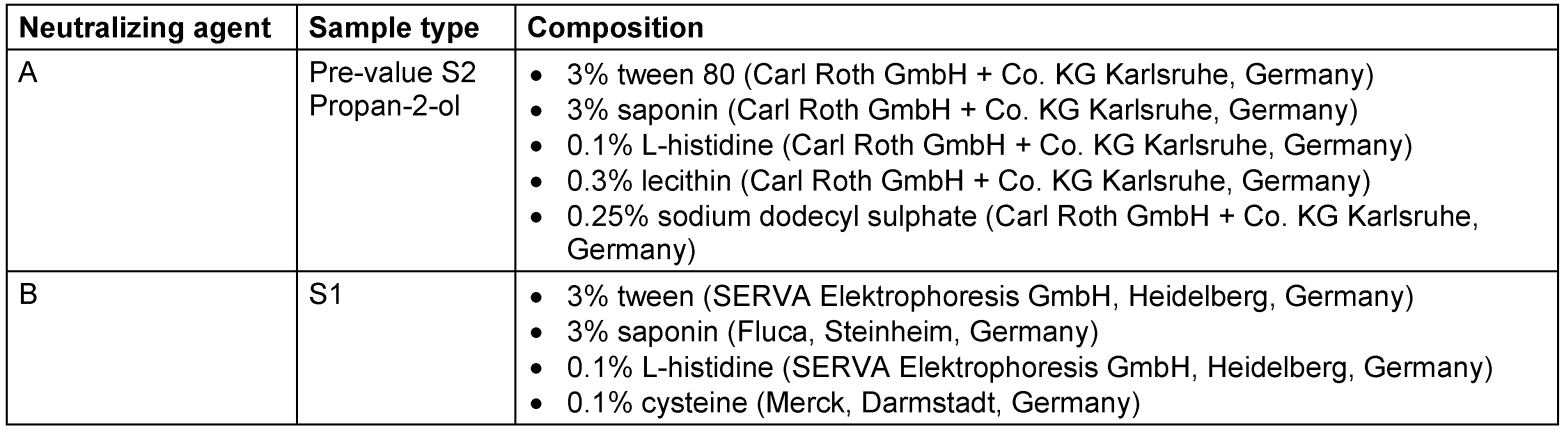
Composition if neutralizing agents used for pre-values, S1, S2 and reference product propan-2-ol

**Table 3:**
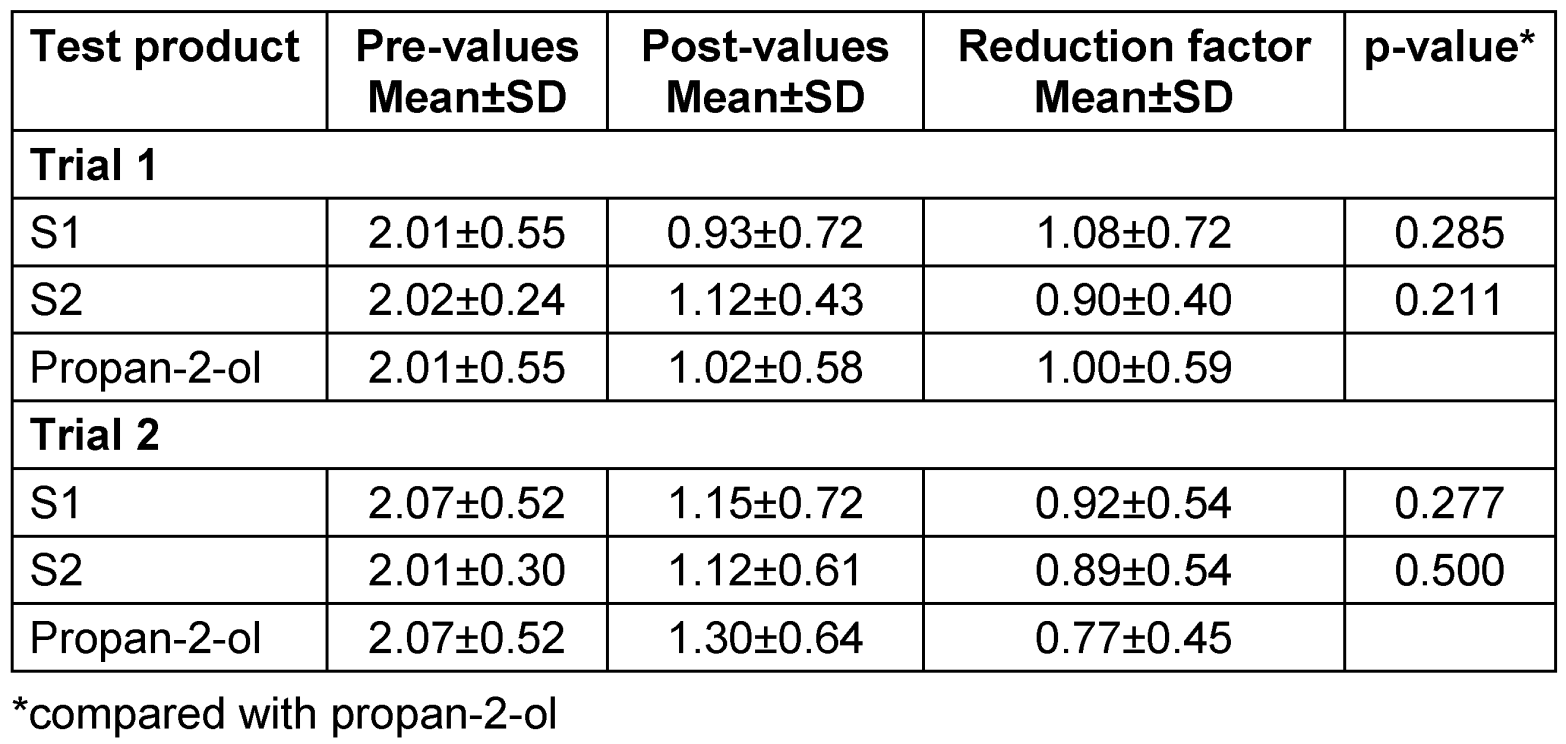
Mean lg pre-values, post-values and reduction with standard deviations (SD) before and after skin swabbing with the test products
